# Quality Response of Two Mini Chinese Cabbage Cultivars to Different Calcium Levels

**DOI:** 10.3390/foods14050872

**Published:** 2025-03-04

**Authors:** Jiaojiao Yang, Jizhong Ma, Wenbin Zhang, Xueqin Gao, Xuehua Wang, Wenxu Chen, Mohammed Mujitaba Dawuda, Wenlin Li, Linli Hu

**Affiliations:** 1 College of Horticulture, Gansu Agricultural University, Lanzhou 730070, China; 2 State Key Laboratory of Aridland Crop Science, Gansu Agricultural University, Lanzhou 730070, China

**Keywords:** *Brassica rapa* ssp. *pekinensis*, calcium levels, tip-burn, nutritional quality

## Abstract

This study investigated the effects of different calcium levels on the nutritional quality and stress resistance of mini Chinese cabbage, focusing on the ‘QYH’ calcium-sensitive cultivar and the ‘HN’ calcium-tolerant cultivar. Plants were treated with five calcium levels (0, 2, 4, 6, and 8 mmol/L) to analyze the incidence of tip-burn, tissue calcium content, mineral accumulation, amino acid composition, and phenolic and flavonoid compound contents. The results showed that appropriate calcium levels significantly reduced tip-burn incidence. Specifically, ‘QYH’ exhibited no tip-burn symptoms at 6 mmol/L calcium, while ‘HN’ was tip-burn free at 4 mmol/L. Appropriate calcium levels also significantly increased the contents of soluble sugars, proteins, and ascorbic acid while reducing nitrate levels in both cultivars. For example, the soluble sugar content in ‘QYH’ increased by 119.05% under the 6 mmol/L calcium treatment. Similarly, ‘HN’ showed significant increases in soluble sugars, proteins, and ascorbic acid at 4 mmol/L. Amino acid and phenolic compound levels peaked at 6 mmol/L calcium in ‘QYH’, with rutin content in ‘QYH’ increasing by 181.58%. In ‘HN’ these compounds peaked at 4 mmol/L. Additionally, high calcium levels did not antagonize key minerals but reduced manganese accumulation. These findings highlight calcium’s critical role in enhancing the nutritional quality of mini Chinese cabbage and provide a scientific basis for optimizing calcium fertilizer application for both ‘QYH’ and ‘HN’ cultivars.

## 1. Introduction

As one of the essential nutrient elements, calcium plays a vital role in the growth and development of plants and is second only to nitrogen in terms of demand [1]. Calcium is involved in many critical physiological processes in plants, can regulate gene expression [2], maintain cell function [3], promote growth and development [4], stabilize plant cell membranes and cell walls [5], and enhance plant resistance to various stresses [6]. Calcium also serves as an important signaling substance in plant signal transduction. For example, it promotes tomato growth under saline and alkaline conditions [7] and enhances plant tolerance to low temperatures [8].

Moreover, calcium has a significant impact on the quality of crops. Its deficiency can lead to metabolic disorders in many crops, causing diseases and reducing product quality. For example, foliar application of 1.5 times the recommended concentration of calcium nitrate solution significantly reduced the incidence of tip-burn in cabbage [9]. The quality of crops depends not only on physical appearance but also on safety and nutritional composition. This includes beneficial compounds such as phenolic substances, soluble sugars, soluble proteins, and ascorbic acid (AsA), as well as essential nutrient elements like potassium (K), calcium (Ca), iron (Fe), and zinc (Zn) [10]. Calcium fertilizers were found to help maintain the visual quality of broccoli and the ascorbic acid content in its flower stems [11]. A higher concentration of calcium increased the phenolic compound content and total antioxidant activity in peach fruits [12], improving fruit nutritional quality, although it reduced titratable acid content [13]. Nitrate content is an important safety indicator for vegetables. Excessive nitrate accumulation in humans, mainly from dietary sources, can lead to health issues such as cancer [14]. Nitrate is one of the primary nitrogen forms absorbed by crops in natural and agricultural systems. It acts as a signaling molecule, regulating gene expression and various processes of plant growth and development [15,16,17]. For instance, the nitrate content in apples was found to decrease as calcium concentrations increased [18]. Calcium deficiency also reduces soluble sugar content in melons [19]. In another experiment, spraying cherries with calcium reduced the phenolic acid and flavonoid contents [20]. Calcium application improved cucumber quality by increasing free amino acid content [21]. Among the essential nutrient elements required by vegetable crops, except for carbon, hydrogen, and oxygen derived from air and water, other elements primarily come from the soil [22]. Potassium (K) and magnesium (Mg) are particularly important for plant growth and development [23]. However, the Mg, P, K, and N contents in tomato fruits tend to decrease with higher calcium concentrations. Exogenous calcium application increased the calcium content in pomegranate leaves and peels but had antagonistic effects on other nutrient elements [24].

Mini Chinese cabbage (*Brassica pekinensis*) belongs to the Brassicaceae family. It is characterized by high yield, good quality, a short growth cycle, and significant economic value. In recent years, calcium deficiency has seriously affected its commercial value [25]. To better understand how different calcium levels affect the quality of mini Chinese cabbage, a pot experiment was conducted to determine the effects of different calcium levels on the quality of calcium-sensitive (refers to the increased calcium demand of plants, making them more susceptible to a low-calcium environment) and calcium-tolerant (refers to the ability of plants to grow and develop normally in a low-calcium environment) mini Chinese cabbage cultivars. This study aims to identify the most suitable calcium fertilizer application rate for improving mini Chinese cabbage quality and provide a theoretical basis for reducing the occurrence of tip-burn through calcium application.

## 2. Materials and Methods

### 2.1. Plant Materials

The calcium-sensitive cultivar ‘QYH’ (Beijing Sihai Seed Industry Co., Ltd., Beijing, China) and calcium-tolerant cultivar ‘HN’ (Beijing Huanai Agricultural Development Co., Ltd., Beijing, China) were used as the experimental materials.

The pot experiment was carried out in the modern greenhouse located at Gansu Agricultural University from June to August 2021. The seeds were sterilized and placed in an artificial climate chamber for germination at 25 °C in the dark. After 18 h of germination, the germinated seeds were sown in 50-hole trays. At the three-leaf stage, uniform seedlings without diseases and insect pest symptoms were transplanted in pots (15 cm high, 25 cm in height, upper circle diameter 25 cm, lower circle diameter 15 cm) with substrates [perlite: vermiculite = 1:3 (*v*:*v*)], and each pot contained one seedling.

### 2.2. Experimental Design

According to the research of Heaney et al. [26], approximately 1.61 kg of elemental calcium is required to produce 1000 kg of Chinese cabbage. Based on the average biological yield of 1.5 kg of mini Chinese cabbage in the experiment, a single mini Chinese cabbage required 2.4 g of pure calcium in the whole growth period, about 60 mmol; about 6 mmol (10%) of calcium is required at the seedling stage, about 18 mmol (30%) at the rosette stage, and about 36 mmol (60%) at the heading stage, which is determined as the normal supply of calcium. According to the above data, the element concentrations in Hoagland’s nutrient solution was referred as described by Wang et al. (except calcium). Five calcium levels (0 mmol/L, 2 mmol/L, 4 mmol/L, 6 mmol/L, 8 mmol/L) were supplied to the seedlings. The calcium concentration of normal Hogland nutrient solution is 5 mmol/L, which is provided by calcium nitrate. When the calcium concentration is lower than 5mmol/L, the missing nitrogen element is provided by ammonium nitrate; when the calcium concentration is higher than 5 mmol/L, the additional calcium is provided by calcium chloride. Table 1 shows the amount of ammonium nitrate and calcium chloride added.

The experiment adopted a completely randomized design. Each treatment was repeated 3 times, with each repetition consisting of 20 pots. The nutrient solution was renewed every 10 d during the seedling stage and at 6 d intervals during vigorous growth. Other field management procedures were the same.

### 2.3. Determination Method

#### 2.3.1. Incidence and Disease Index of Tip-Burn

On the 40th d after transplant, the leaf edge of mini Chinese cabbage began to show the symptoms of tip-burn. The disease index of tip-burn was observed once every 5 d until harvest. The classification standard for tip-burn symptoms was based on Table 2. The disease index of tip-burn was calculated by the following formula according to the method of Li et al. [25].

Disease index = Σ (serial number × number of diseased plants)/(highest level × total number of plants) × 100

#### 2.3.2. Mineral Element

Mini Chinese cabbage was harvested and placed in a DHG-9141A drying oven (Shanghai Yiheng Scientific Instrument Co., Ltd., Shanghai, China), dried at 105 °C for 30 min, and then at 80 °C to constant weight. The dried samples were ground and sieved through a sieve with a pore size of 0.25 mm. The dried samples were pretreated for measuring mineral element by the wet digestion method (H_2_SO_4_-H_2_O_2_).The molybdenum-antimony anti-colorimetric method was used for phosphorus (P) element determination, the K9860 Kjeldahl nitrogen analyzer (Haineng K1100) was used for nitrogen (N) determination, and the determination of metal elements potassium (K), calcium (Ca), magnesium (Mg), copper (Cu), iron (Fe), manganese (Mn), and zinc (Zn) were referred to the method [27] with an AFG atomic absorption spectrophotometer (Jena, Germany, ZEEnit 700P).

#### 2.3.3. Soluble Sugar, Soluble Protein, Ascorbic Acid, Titratable Acid, Nitrate Content

At the end of the heading stage, five mini Chinese cabbage leafy balls were randomly selected from each treatment, and a quarter of each leaf ball was chopped and mixed for sampling. The inner leaves of cabbage were taken from the treatment without the ball for the following quality determination. The content of soluble sugar was measured by anthrone colorimetry [28]. The content of soluble protein was measured by the Coomassie Brilliant Blue G-250 staining method [29]. The AsA content was measured by the 2,6-dichlorostarch sodium staining method [30]. The titratable acid was determined by the acid–base titration method [31]. The nitrate content was determined by the nitrosalicylic acid method [31].

#### 2.3.4. Amino Acid Components

The 0.5 g dried leaf was accurately weighed and put in a conical flask, then 20 mL of 0.1% hydrochloric acid solution was added for ultrasonic extraction for 15 min (25 °C), then the extract was transferred to a 10 mL centrifuge tube and centrifuged at 10,000 r/min for 5 min, and a syringe was used to take the supernatant through a 0.22 μm aqueous phase filter membrane for testing.

According to the LC-MS method, the chromatographic conditions were as follows: XTerra MS C18 (50 mm × 2.1 mm × 2.5 μm), the injection volume was 5 μL, the column temperature was 25 °C, the mobile phase A was 0.1% formic acid; the mobile phase B was acetonitrile, and the elution condition was A:B = 95:5. The flow rate was 0.5 mL/min.

According to the methods of Sams and Conway [32], with minor modification, amino acid components were classified into the following categories: essential amino acids (EAAs), non-essential amino acids (NEAAs), and semi-essential amino acids (cEAAs).

#### 2.3.5. Phenolic Acids and Flavonoids

Leaf samples were quickly put into liquid nitrogen, then frozen in a −80 °C refrigerator for 24 h, followed by vacuum low-temperature freeze-drying, and then they were taken out after 36 h and ground into powder and put into a −80 °C refrigerator for later use. Dry powder weighing 0.1 g of was added to 2 mL of methanol, placed at room temperature for 1 h, centrifuged at 4 °C and 8000 rpm for 10 min, and the supernatant was filtered with a 0.22 μm filter membrane for determination.

The HPLC analysis of polyphenols was carried out using a Waters liquid chromatography system (e2695, Waters, with 2998 PDA detector) equipped with a 1525 pump and a 2998 photodiode array detector. HPLC 18 column (250 mm × 4.6 mm, Waters Symmetry); column temperature: 30 °C; flow rate: 1.1 mL min^−1^; mobile phase: methanol (A), 1% acetic acid (B) Using gradient elution, pipette 10 μL of the sample, detect p-hydroxybenzoic acid, protocatechuic acid, quercetin, chlorogenic acid, and rutin at 240 nm, detect cinnamic acid, gallic acid, naringenin, and benzoic acid at 280 nm, and ferulic acid, mesonic acid, caffeic acid, cybinin, and gentisic acid were detected at 322 nm.

All chemical standards used were purchased from Beijing Solarbio Technology Co., Ltd., Beijing, China, with a purity of HPLC ≥ 95%.

### 2.4. Data Analysis

Microsoft Excel 2010 software was used to analyze data. Origin 2022 was used to draw graphs. SPSS 20 Software was used to perform two-way analysis of variance (ANOVA) with Tukey’s test for multiple comparisons, with a significance level set at *p* < 0.05. Origin 2022 was used for principal component analysis (PCA).

## 3. Results

### 3.1. Effects of Different Calcium Levels on the Incidence and Disease Index of Leaf Tip Burns in Mini Chinese Cabbage

**Figure 1:**
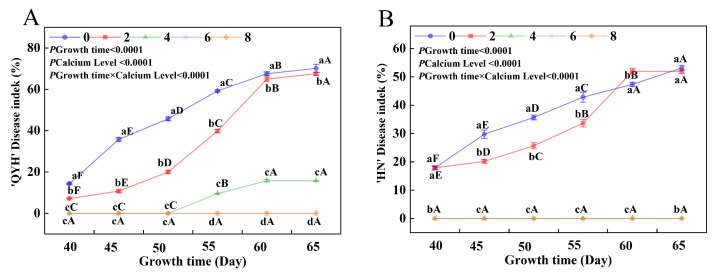
Effect of calcium levels on the tip-burn disease index of two cultivars of mini Chinese cabbage. The disease indexes of ‘QYH’ (**A**) and ‘HN’ (**B**) at different calcium levels. Data were expressed as mean ± SD (n = 20 seedlings from three independent experiments). Different lowercase letters indicated significant differences between different calcium levels within the same cultivar (*p* < 0.05), and different uppercase letters indicated significant differences between different growth times at the same calcium level (*p* < 0.05). Based on Tukey’s test, the values after *p* growth time × calcium level represented the significance of the interaction after two-way analysis.

With the extension of growth period, the disease indices of the ‘HN’ and ‘QYH’ also increase. However, no significant difference in soluble sugar content was found between 6 mmol/L and 8 mmol/L for the ‘HN’ cultivar cultivars treated with 0 and 2 mmol/L showed an increasing trend (Figure 1A,B). At 40 days, both cultivars began to develop disease symptoms under 0 and 2 mmol/L calcium levels. On day 45, ‘QYH’ incidence was higher than ‘HN’, the difference was 3.4, but not significant. However, from day 55 onwards, the disease index of ‘QYH’ was already significantly higher than that of ‘HN’, with a difference of 20.1 by 60 d. This demonstrated that ‘QYH’ has greater sensitivity to calcium deficiency. Under the 2 mmol/L calcium treatment, both cultivars showed an increasing disease index over time. While ‘HN’ had a slightly higher disease index at 40 d, with ‘QYH’ exhibiting a significantly higher disease index from 55 d onward. Interestingly, ‘QYH’ treated with 4 mmol/L calcium also began to show disease symptoms from the 50th day, suggesting that even this calcium level was not entirely sufficient to prevent disease development in this variety. In contrast, ‘HN’ at 4 mmol/L remained largely symptom-free throughout the experiment. These results clearly indicated that ‘QYH’ was more sensitive to calcium deficiency than ‘HN’.

### 3.2. Ca Content in Different Tissues of Mini Chinese Cabbage in Different Periods

#### 3.2.1. Variance Analysis

**Figure 2:**
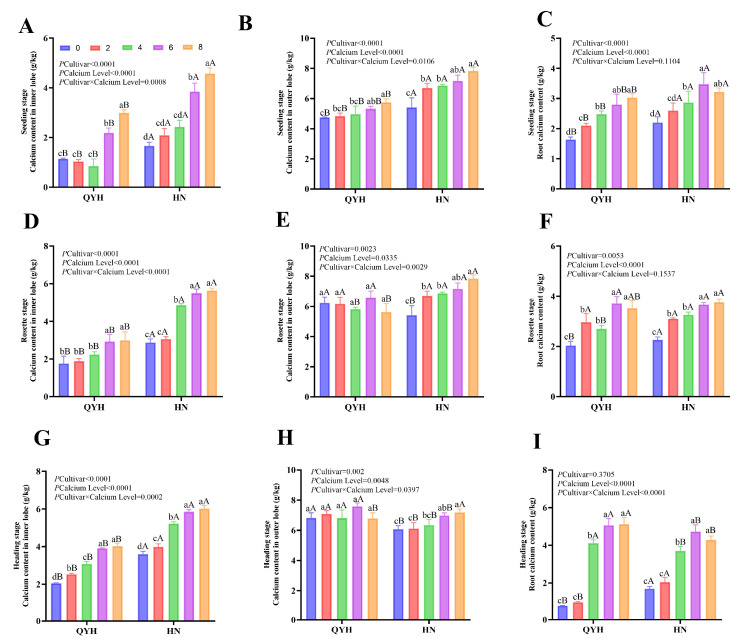
The calcium content in the inner leaves (**A**), outer leaves (**B**), and roots (**C**) during the seedling stage, inner leaves (**D**), outer leaves (**E**), and roots (**F**) during the rosette stage, and inner leaves (**G**), outer leaves (**H**), and roots (**I**) during the heading stage of mini Chinese cabbage under different calcium level treatments. Data were presented as mean ± SD (n = 3) from three independent replications. Different lowercase letters indicated significant differences between calcium levels within the same cultivar (*p* < 0.05), and different uppercase letters indicated significant differences between cultivars at the same calcium level (*p* < 0.05). Based on Tukey’s test, the values following *p* cultivar × calcium level represented the significance of the interaction after two-way analysis.

The Ca contents in different organs were different under different Ca levels, which showed an increasing trend throughout the growth period (Figure 2). The Ca content of inner leaves under 6 mmol/L and 8 mmol/L calcium levels was significantly higher than those of the other treatments (Figure 2A,D,G) and increased by 66.48% and 69.89% in the rosette stage compared with the 0 mmol/L and 2 mmol/L treatments, respectively. Moreover, the Ca content in the inner leaves of ‘HN’ was 1.88 times higher than that of ‘QYH’. The Ca content in the outer leaves was higher than that in the inner leaves, and the change range among different growth stages was not large (Figure 2B,E,H). Moreover, the Ca content reached the highest value at 8 mmol/L; ‘QYH’ increased by 20.98% and ‘HN’ by 44.88% compared to the calcium-free treatment at the seedling stage (Figure 2B). Under 8 mmol/L treatment, ‘HN’ increased by 30.77% compared with 0 mmol/L treatment at the rosette stage, and ‘QYH’ had no significant difference in calcium levels. In roots, ‘HN’ generally accumulated more Ca than ‘QYH’ (Figure 2C,F,I). While both cultivars reached the highest root Ca content at 8 mmol/L, there was no significant difference between the 6 mmol/L and 8 mmol/L treatments. However, both cultivars showed a significant decrease in root Ca content at 0 and 2 mmol/L during the heading stage. The root calcium content of ‘HN’ was generally higher than that of the ‘QYH’ variety, which reached the highest contents at 8 mmol/L but had no significant difference with the 6 mmol/L treatment. However, the contents of 0 mmol/L and 2 mmol/L decreased significantly during the heading stage (Figure 2C).

#### 3.2.2. Principal Component Analyses (PCA)

**Figure 3:**
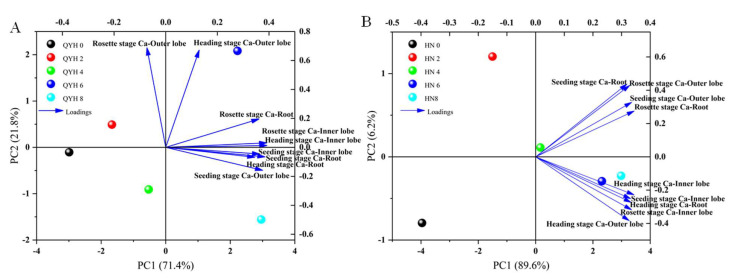
Principal component analysis (PCA) of mini Chinese cabbage under different calcium level treatments at different stages for QYH cultivar (**A**) and HN cultivar (**B**). Data are presented as mean values (n = 3). Blue arrows indicated the direction of the distribution of substances within the quadrant.

The models based on PCA (Principal Component Analysis) for the effects of different calcium levels on the calcium content in mini Chinese cabbage are shown in Figure 3A (‘QYH’ cultivar) and Figure 3B (‘HN’ cultivar). In the ‘QYH’ cultivar, the first two principal components accounted for 85.4% of the variance, with PC1 and PC2 explaining 71.4% and 21.8% of the total variance, respectively. The loading plot revealed that calcium content in the inner leaves during the rosette and heading stages contributed most to PC1, while calcium content in the outer leaves during the rosette and heading stages contributed most to PC2. These factors can serve as representative indicators of calcium content levels in different growth stages and plant parts of the QYH cultivar.

In the ‘HN’ cultivar (Figure 3B), the first two principal components accounted for 95.8% of the variance, with PC1 and PC2 explaining 89.6% and 6.2% of the total variance, respectively. The loading plot showed that calcium content in the rosette roots contributed most to PC1, while calcium content in the seedling roots contributed most to PC2. These factors can serve as representative indicators of calcium content levels in different growth stages and plant parts of the HN cultivar.

A clear separation of treatments based on PC1 and PC2 was observed, with HN-6 and HN-8 located in the same quadrant, suggesting similar patterns between these treatments.

### 3.3. The Effect of Different Calcium Levels on the Element Level of Mini Chinese Cabbage

#### 3.3.1. Variance Analysis

**Figure 4:**
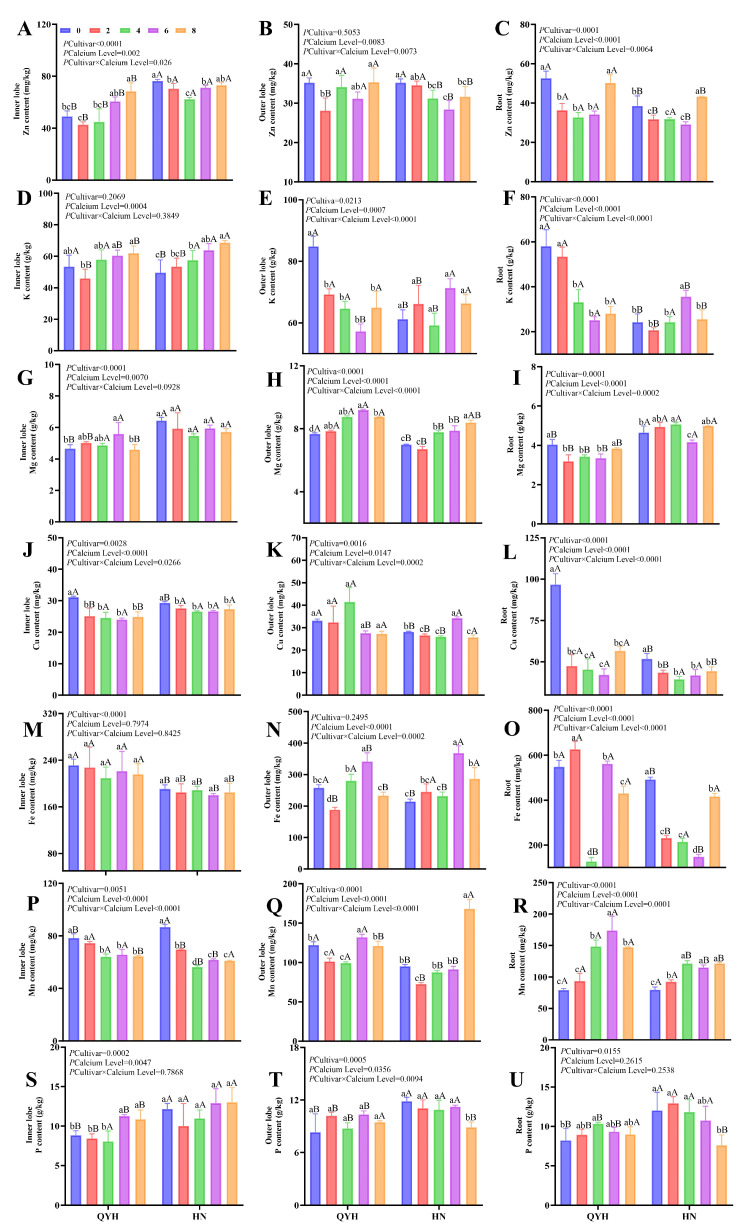
The element contents in mini Chinese cabbage under different calcium level treatments. Inner leaf Zn (**A**), outer leaf Zn (**B**), root Zn (**C**); inner leaf K (**D**), outer leaf K (**E**), root K (**F**); inner leaf Mg (**G**), outer leaf Mg (**H**), root Mg (**I**); inner leaf Cu (**J**), outer leaf Cu (**K**), root Cu (**L**); inner leaf Fe (**M**), outer leaf Fe (**N**), root Fe (**O**); inner leaf Mn (**P**), outer leaf Mn (**Q**), root Mn (**R**); inner leaf P (**S**), outer leaf P (**T**), root P (**U**). Data are presented as mean ± SD (n = 3) from three independent replicates. Different lowercase letters indicate significant differences between calcium levels within the same cultivar (*p* < 0.05), and different uppercase letters indicate significant differences between cultivars at the same calcium level (*p* < 0.05). Based on Tukey’s test, the values following *p* cultivar × calcium level represented the significance of the interaction after two-way analysis.

In mini Chinese cabbage, the impact of calcium levels on element content varied between different plant organs, including the inner leaves, outer leaves, and roots (Figure 4).

Calcium application significantly influenced the mineral content of inner leaves, though the response varied between cultivars. The Zn content in ‘QYH’ reached the highest value at 8 mmol/L calcium level, while that in ‘HN’ occurred at 0 mmol/L calcium level, but there was no significant difference with the 8 mmol/L calcium level (Figure 4A). The K content in the inner leaves of ‘QYH’ did not show significant changes with increasing calcium levels (Figure 4D). The Mg content in the inner leaves of ‘QYH’ was highest at 6 mmol/L calcium, increasing by 20% compared to the 0 mmol/L calcium treatment, while no significant differences were observed in the ‘HN’ cultivar across different calcium levels (Figure 4G). The Fe content in the inner leaves did not show significant variation with different calcium treatments (Figure 4M), indicating that calcium did not affect iron accumulation in the inner leaves. At 6 mmol/L and 8 mmol/L calcium levels, the phosphorus content in the inner leaves of mini Chinese cabbage was the highest, significantly higher than in the outer leaves and roots (Figure 4S–U). This suggests that higher calcium levels promote the distribution of phosphorus towards the inner leaves.

In the outer leaves, zinc content showed similar patterns to the inner leaves but was generally lower in comparison (Figure 4B). The K content was higher in the outer leaves than in the inner leaves and root, following the order of outer leaves > inner leaves > root (Figure 4D–F). Under treatments of 4, 6, and 8 mmol/L calcium, potassium content in the root system was significantly lower than in the outer leaves. For Mg, the outer leaves of ‘QYH’ had higher magnesium content than those of ‘HN’. The magnesium content in the outer leaves of ‘QYH’ was highest at the 6 mmol/L calcium level, while in ‘HN’, the highest magnesium content in the outer leaves was observed at 8 mmol/L calcium (Figure 4H). The Mn content in the outer leaves initially increased with calcium levels before decreasing at higher calcium concentrations, with a significant decrease at 8 mmol/L (Figure 4Q). The higher potassium and magnesium content in the outer leaves compared to other organs highlights their role in mineral nutrient accumulation.

In the roots, Zn content followed a similar trend to that of the inner leaves, but with generally lower values (Figure 4C). K content in the root system was the lowest of all organs, and under the treatments of 4, 6, and 8 mmol/L calcium, the potassium content in the roots was significantly lower than in the inner and outer leaves (Figure 4F). For Mg, the root system exhibited lower levels than the leaves, with the magnesium content in the roots of both cultivars highest at 8 mmol/L calcium, though no significant differences were observed between 6 and 8 mmol/L treatments (Figure 4I). Cu content in the roots was highest at 0 mmol/L calcium, and it decreased with increasing calcium levels, indicating that calcium has an antagonistic effect on copper absorption by the root system (Figure 4L). The Mn content in the roots was significantly higher at 4, 6, and 8 mmol/L calcium levels compared to the 0 and 2 mmol/L levels (Figure 4R). Additionally, the P content in the roots did not show a marked trend under different calcium treatments (Figure 4U). These findings suggest that while the root system shows an overall lower uptake of most nutrients, calcium treatments still influence the absorption of specific elements such as copper and manganese.

#### 3.3.2. Principal Component Analyses (PCA)

**Figure 5:**
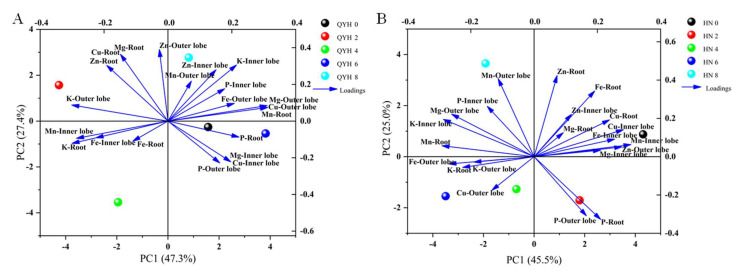
Principal component analysis of element content in mini Chinese cabbage under different calcium level treatments. ‘QYH’ cultivar (**A**), ‘HN’ cultivar (**B**). Data are presented as mean values (n = 3). Blue arrows indicate the direction of the distribution of substances within the quadrant.

The models based on PCA for the effects of different calcium levels on the elemental content in mini Chinese cabbage are shown in Figure 5A (‘QYH’ cultivar) and Figure 5B (‘HN’ cultivar). In ‘QYH’ cultivar, the first two principal components accounted for 74.7% of the variance, with PC1 and PC2 explaining 47.3% and 27.4% of the total variance, respectively. The first principal component was strongest for Mg-Outer lobe, Cu-Outer lobe, and Mn-Root, while the second principal component was strongest for K-Inner lobe. The different calcium treatments showed clear separation in the PCA plot, indicating that calcium levels significantly affect the elemental content.

In ‘HN’ cultivar, PC1 and PC2 explained 45.5% and 25.0% of the variance, respectively, with both components together accounting for 70.5% of the total variance. Zn-Outer lobe and Mn-Inner lobe had the strongest first principal component, while Zn-Root had the strongest second principal component. These factors can serve as representative indicators of the impact of different calcium levels on the elemental content in mini Chinese cabbage. Additionally, each treatment showed significant separation based on PC1 and PC2.

### 3.4. Effects of Different Calcium Levels on the Quality of Mini Chinese Cabbage

#### 3.4.1. Variance Analysis

**Figure 6:**
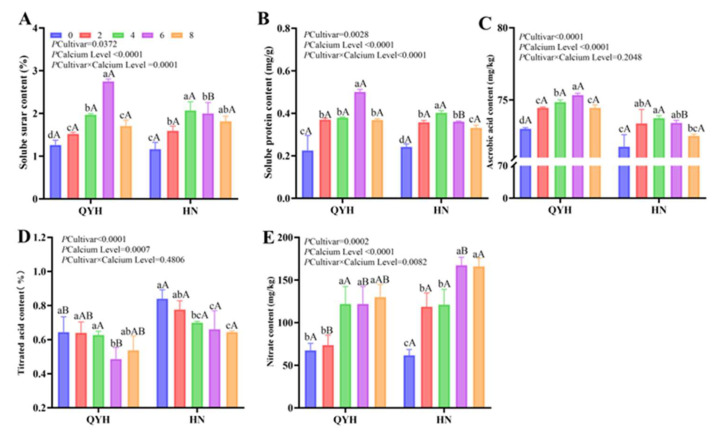
Quality indicators of mini Chinese cabbage under different calcium treatments, (**A**) Soluble sugar content, (**B**) Soluble protein content, (**C**) AsA content, (**D**) Titrated acid content, (**E**) Nitrate content. Data are presented as mean ± SD (n = 3) from three independent replicates. Different lowercase letters indicate significant differences between calcium levels within the same cultivar (*p* < 0.05), and different uppercase letters indicate significant differences between cultivars at the same calcium level (*p* < 0.05). Based on Tukey’s test, the values following *p* cultivar × calcium level represented the significance of the interaction after two-way analysis.

The soluble sugar content of ‘QYH’ was highest at 6 mmol/L, which was 2.76%, given an increase of 119.05% compared with 0 mmol/L calcium level. Under the conditions of no calcium (0 mmol/L) and low calcium (2 mmol/L), the soluble sugar contents of the two cultivars of mini Chinese cabbage were significantly lower than the calcium level of 4, 6 and 8 mmol/L. For the ‘QYH’ cultivar, a significant difference in soluble sugar content was observed between 6 mmol/L and 8 mmol/L, with 6 mmol/L resulting in a higher value. However, no significant difference in soluble sugar content was found between 6 mmol/L and 8 mmol/L for the ‘HN’ cultivar. (Figure 6A). The content of soluble protein showed a trend of an initial increase and then a decrease with the increase of calcium level (Figure 6B), and the content in ‘QYH’ reached the highest value at 6 mmol/L calcium level, while that in ‘HN’ reached the maximum value at 4 mmol/L calcium level. Moderately increasing the calcium level could significantly increase the ascorbic acid content of mini Chinese cabbage (Figure 6C).The titratable acid content of ‘HN’ cultivar ranged from 0.64 to 0.84 mg/g (Figure 6D), while that of ‘QYH’ cultivar ranged from 0.49 to 0.64 mg/g, and the content in ‘HN’ was slightly higher than that in ‘QYH’, the titratable acid content of ‘HN’ showed a decreasing trend with increasing calcium levels, while that of ‘QYH’ showed a downward trend at 6 mmol/L and 8 mmol/L calcium levels, but there was no significant difference between the two.

Calcium level has a significant effect on the nitrate content (Figure 6E) in the two cultivars, and the nitrate content is sensitive to the change of calcium level. The nitrate in both cultivars was the lowest at 0 mmol/L calcium level. With the increase of calcium level, the nitrate content increased, and both cultivars reached the maximum value at 8 mmol/L calcium levels, but there was no significant difference between 4, 6 mmol/L calcium levels in ‘QYH’, and there was no significant difference between 6 mmol/L calcium levels in ‘HN’.

#### 3.4.2. Principal Component Analyses (PCA)

**Figure 7:**
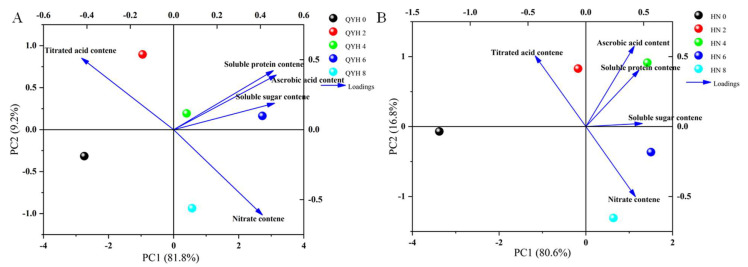
Principal component analysis of quality indicators of mini Chinese cabbage under different calcium treatments. QYH cultivar (**A**), HN cultivar (**B**). Data are presented as mean values (n = 3). Blue arrows indicate the direction of the distribution of substances within the quadrant.

PCA was performed on the quality traits to assess the effects of different calcium levels on mini Chinese cabbage, with the scores and loading plots shown in Figure 7. In ‘QYH’ cultivar (Figure 7A), PC1 explained 81.8% of the variance, and PC2 explained 9.2%, with both components together accounting for 91% of the total variance. The titratable acid content and soluble protein content had strong negative loadings on the first principal component, while soluble sugar content had strong loadings on the second principal component. In the ‘HN’ cultivar (Figure 7B), PC1 explained 80.6% of the variance and PC2 explained 16.8%, with both components together accounting for 97.4% of the total variance. Soluble protein content and soluble sugar content contributed most to PC1, while titratable acid contributed most to PC2.

At the same time, different calcium treatments also showed clear separation in the PCA score plot. Additionally, treatments QYH-4 and QYH-6, as well as HN-6 and HN-8, were located in the same quadrant, indicating similar patterns between these treatments.

### 3.5. Effects of Different Calcium Levels on Amino Acid Composition of Mini Chinese Cabbage

#### 3.5.1. Variance Analysis

A total of 21 free amino acids (FAAs) were detected, including 4 semi-essential amino acids (cEAAs), 8 essential amino acids (EAAs), and 9 non-essential amino acids (NEAAs). Amino acid content was greatly affected by calcium level, among which the content of semi-essential amino acids arginine, histidine, and tyrosine were consistent in the two cultivars, with the increase of calcium level showing a trend of an initial increase and then a decrease. The ‘HN’ cultivar had the highest at 4 mmol/L calcium level, which were 69.43%, 161.73%, and 79.99% higher than those under 0 mmol/L calcium level, respectively. The content of cysteine in ‘HN’ was the highest at the calcium level of 6 mmol/L (Table 3). The calcium level of 6 mmol/L significantly increased the content of semi-essential amino acids in the leaf of ‘QYH’, and the contents of the four amino acids were consistent. When the calcium level increased to 8 mmol/L, the contents of the four amino acids all decreased, indicating that an excessively high level of calcium supply would inhibit the accumulation of semi-essential amino acids in mini Chinese cabbage.

Among the essential amino acids, the lysine content was the highest (Table 4). The ‘HN’ cultivar had the highest content of 13,092.99 mg/Kg under 4 mmol/L calcium level, an increase of 91.68% compared with that under 0 mmol/L calcium level. While the content was the highest at 6 mmol/L calcium level in ‘QYH’ cultivar, 14,529.76 mg/Kg, an increase of 208.96% compared with 0 mmol/L calcium level. Among the ‘QYH’ cultivars, except for threonine and phenylalanine, the contents of the other six essential amino acids were the highest at 6 mmol/L calcium level, while all the eight essential amino acid contents in ‘HN’ reached the highest value at 4 mmol/L calcium level and decreased significantly at the levels of 6 mmol/L and 8 mmol/L, indicating that the 4 mmol/L calcium level was the most favorable for the accumulation of essential amino acids in ‘HN’, and the 6 mmol/L calcium level was the most favorable for accumulation of essential amino acids in ‘QYH’ cultivar.

Among the non-essential amino acids in mini Chinese cabbage, the highest content was aspartic acid, while the lowest content was cystine (Table 5). In the ‘HN’ cultivar, for asparagine, proline, glycine, serine, and aspartic acid, there was no significant difference between the 4 mmol/L calcium level and 8 mmol/L calcium level, indicating that high calcium supply had no significant effect on the accumulation of these five non-essential amino acids. The lower content was significantly higher than other treatments, indicating that high calcium is beneficial to the accumulation of these two amino acids. In the ‘QYH’ cultivars, except for cystine, the contents of the other eight non-essential amino acids were the highest under 6 mmol/L calcium level, and the contents of alanine, glycine, and glutamic acid were 22,500.02 mg/Kg, 1104.94 mg/Kg, and 7285.93 mg/Kg, respectively, decreased by 19.39%, 17.48%, and 22.36% compared with the 8 mmol/L calcium level, indicating that the 8 mmol/L calcium level is not conducive to the accumulation of non-essential amino acids in the ‘QYH’ cultivar.

#### 3.5.2. Principal Component Analysis (PCA)

**Figure 8:**
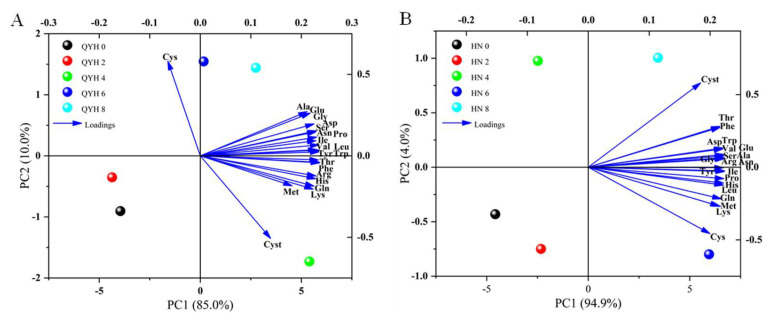
Principal component analysis of amino acid profiles of mini Chinese cabbage under different calcium treatments. (**A**) QYH cultivar; (**B**) HN cultivar. Data are presented as mean values (n = 3). Blue arrows indicate the direction of distribution for each amino acid within the quadrant.

PCA of phenolic acids and flavonoids in mini Chinese cabbage under different calcium levels is shown in Figure 8. In the ‘QYH’ cultivar (Figure 8A), PC1 explained 85.0% of the variance, while PC2 explained 10.0%, with both components together accounting for 95.0% of the total variance. The loading plot revealed that the first principal component was strongest for amino acids such as Tyrosine, Tryptophan, Valine, and Leucine. The second principal component was strongest for amino acids such as Alanine and Glutamic acid.

In the ‘HN’ cultivar (Figure 8B), PC1 explained 94.9% of the variance, and PC2 explained 4.9%, with both components together accounting for 99.8% of the total variance. This indicated that the two principal components could explain most of the data variability. The loading plot showed that the first principal component was strongest for amino acids such as Arginine, Asparagine, Serine, Alanine, Valine, and Glutamic acid, while the second principal component was strongest for Cysteine. These factors can serve as representative indicators of the impact of different calcium levels on amino acid content. Based on the separation of treatments along PC1 and PC2, different calcium levels had a significant effect on the amino acid composition of mini Chinese cabbage.

### 3.6. Effects of Different Calcium Levels on Phenolic Acids and Flavonoids in Pakchoi 

#### 3.6.1. Variance Analysis

**Figure 9:**
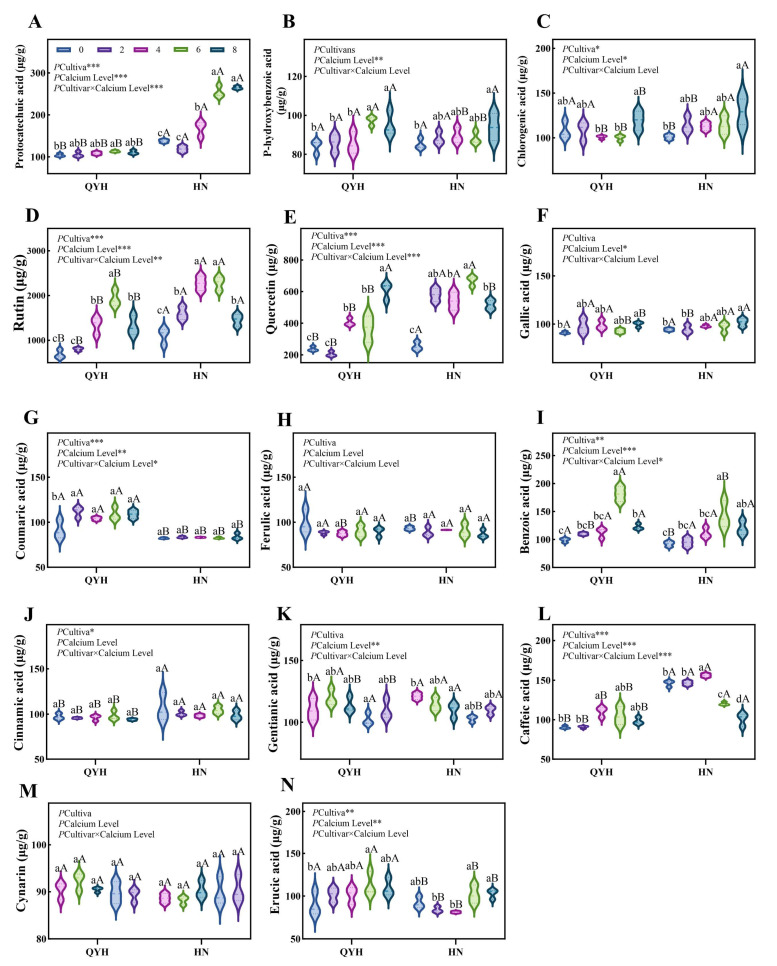
Phenolic acid flavonoids contents in mini Chinese cabbage under different calcium levels. (**A**) Protocatechuic acid, (**B**) p-Hydroxybenzoic acid, (**C**) Chlorogenic acid, (**D**) Rutin, (**E**) Quercetin, (**F**) Gallic acid, (**G**) Coumaric acid, (**H**) Ferulic acid, (**I**) Benzoic acid, (**J**) Cinnamic acid, (**K**) Gentianic acid, (**L**) Caffeic acid, (**M**) Cynarin, (**N**) Erucic acid. Data are presented as mean ± SD (n = 3) from three independent replicates. Different lowercase letters indicate significant differences between calcium levels within the same cultivar (*p* < 0.05), while different uppercase letters indicate significant differences between cultivars at the same calcium level (*p* < 0.05). Based on Tukey’s test. Asterisks (*, **, ***) denote significant differences at *p* < 0.05, *p* < 0.01, and *p* < 0.001 levels, respectively (two-way analysis).

A total of 14 kinds of phenolic acids and flavonoids were detected in our experiment (Figure 9), including 12 kinds of phenolic acids and 2 kinds of flavonoids. The 12 phenolic acids were protocatechuic acid, p-hydroxybenzoic acid, chlorogenic acid, gall acid, coumaric acid, ferulic acid, benzoic acid, cinnamic acid, gentisic acid, caffeic acid, cynarin, and mesonic acid, and the two flavonoids are quercetin and rutin, respectively. The content of phenolic flavonoids in the two cultivars had little difference. The phenolic flavonoids in ‘QYH’ cultivar was in the range of 2067.52 μg/g to 3604.20 μg/g DW, and the content in ‘HN’ cultivar was in the range of 2582.29 μg/g to 4344.02 μg/g. Within the range, the content of phenolic flavonoids in the two cultivars showed a trend of first increasing and then decreasing with the increase of calcium level, and the content was the highest at 6 mmol/L calcium level, and too high or too low calcium levels will lead to less phenolic flavonoid content. The content of various phenolic acids in the two kinds of mini Chinese cabbage was not high, and the content of the two flavonoids was significantly higher than that of phenolic acids. Among the flavonoids, the content of rutin was the highest, followed by the content of quercetin. The contents of rutin both in ‘QYH’ and ‘HN’ cultivars were the highest at the 6 mmol/L calcium level, which were 181.58% and 106.01% higher than the 0 mmol/L calcium level. The content of 8 mmol/L calcium decreased by 31.50% and 35.97%, respectively, indicating that the appropriate calcium concentration is beneficial to the accumulation of phenolic flavonoids in mini Chinese cabbage and too high or too low is not conducive to its synthesis.

#### 3.6.2. Principal Component Analyses (PCA)

**Figure 10:**
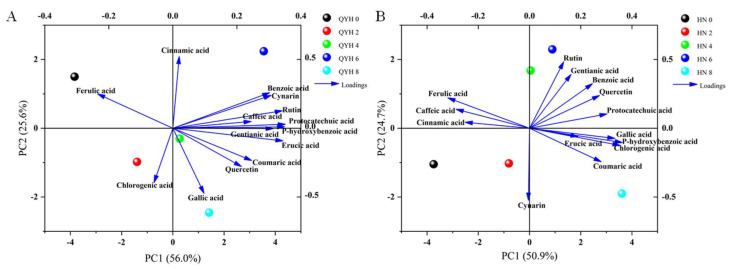
Principal component analysis of phenolic acid flavonoids contents in mini Chinese cabbage under different calcium levels. (**A**) QYH cultivar; (**B**) HN cultivar. Data are presented as mean values (n = 3). Blue arrows indicate the direction of distribution for each phenolic acid flavonoids within the quadrant.

PCA showed that in the ‘QYH’ cultivar (Figure 10A), PC1 explained 56.0% of the variance, while PC2 explained 25.6%, with both components together accounting for 81.6% of the total variance. The first principal component of Protocatechuic acid and p-Hydroxybenzoic acid was the strongest, while the second principal component for Cinnamic acid was the strongest. Therefore, these can be considered representative factors reflecting the phenolic acid and flavonoid content in mini Chinese cabbage under different calcium levels.

In the ‘HN’ cultivar (Figure 10B), the two principal components explained a total variance contribution of 74.9%, with PC1 and PC2 explaining 50.9% and 24.0% of the total variance, respectively. Protocatechuic acid exhibited the strongest first principal component, while Rutin showed a relatively strong second principal component. Additionally, HN-4 and HN-6 were located close to each other, indicating that the phenolic acid composition in these two treatments was similar.

## 4. Discussion

Calcium deficiency in plants is one of the main factors affecting yield and quality, as observed in cabbage by other authors [32], in cucumber [33], and in garlic [34]. Calcium deficiency leads to plant dwarfing, stem tip burning, exposure to biotic stresses, inability to maintain cell wall stability, and loss of quality, whereas calcium deficiency induced stem tip burning is due to necrosis of plant tissues after stress, leading to loss of quality [25], and the cause of stem tip burning varies considerably in different types of vegetables. There are significant differences in the incidence and frequency of occurrence, and their performance characteristics are different [34] and influenced by both genetic and environmental factors.

In this experiment, the inner leaves of the two mini Chinese cabbage cultivars showed significant variability in calcium uptake, and there was no significant change in the calcium content of the outer leaves with the increase in calcium application, and there were some differences in the resistance of different cultivars of cabbage to tip-burn. This is consistent with the results of Yuan et al. [35], who also studied tip-burn in Chinese cabbage. Whereas calcium, which is difficult to mov in the plant, is firstly manifested in the heart leaves, calcium deficiency reduces the accumulation of calcium in the heart leaves, which triggers the occurrence of tip-burn disease. The overall trend of calcium content in leaves increased up to 8 mmol/L. However, in the no and low calcium treatments, the root calcium content in the rhizome stage was much lower than those in the seedling and rosette stages due to incomplete root development or necrosis caused by prolonged calcium deficiency. This observation agrees with an earlier report [36].

K, Mg, Fe, Mn, Zn, and Cu are essential macronutrients for plant growth and development [37]. Studies have shown that their deficiency inhibits plant growth and development and reduces quality [38]. K exists in plants in ionic form and promotes nitrogen metabolism, improves plant water use efficiency and stress tolerance [39], while Mg is a constituent of chlorophyll [40]. In this study, it was found that exogenous application of calcium has an antagonistic effect on the uptake of K and Mg elements in plants [41]. Specifically, calcium application had an antagonistic effect on the uptake of Mg in the root system of ’QYH’ but did not significantly affect the Mg content of the inner leaves of ’HN’. The Cu and Fe contents varied significantly among different cultivars. Additionally, calcium was found to have an antagonistic effect on Cu accumulation in the inner leaves, which could be reduced at calcium levels of 4–6 mmol/L. The Mn content was also significantly reduced.

Studies have shown that the level of calcium application affects the absorption and metabolism of other nutrients in plants [42] and also plays an important role in fruit firmness and soluble sugar content [24]. This is consistent with our findings, which shows that calcium application increase the content of soluble sugars and soluble proteins in the plant, following a general trend of first increasing and then decreasing. However, the changes in the ’QYH’ cultivar were more pronounced, likely due to its greater sensitivity to calcium. Increasing calcium concentration significantly increased the content of ascorbic acid, and the content of ’QYH’ was slightly higher than that of ’HN’, which was consistent with the findings of Lee and Kim [43]. However, the content of titratable acid gradually decreased and was significantly lower under high calcium concentration treatment than no calcium treatment. This is in agreement with the findings of White and Broadley in Chinese cabbage [44]. In this experiment, when the calcium concentration was increased to 8 mmol/L, the content of soluble sugar, soluble protein, and ascorbic acid in the plant decreased to a certain extent, which may be attributed to the fact that the high concentration of calcium caused the plant to be unable to grow and develop normally, which is not conducive to the improvement of the quality of mini Chinese cabbage. This is consistent with the results of Santamaria [14].

Nitrate content, as one of the important safety qualities of vegetables, mainly enters the human body through the consumption of vegetables [17] and is one of the pathways of nitrogen uptake by crops in nature and agricultural systems [45], and the results of the study showed that there is an interaction between calcium and nitrate metabolism pathway [46], and in the results of the present experiments, the nitrate content tended to increase with the increase of calcium concentration, and the high-calcium treatment was significantly higher than the no-calcium treatment. Leafy vegetables are nitrogen-loving crops and the increase in calcium ion concentration caused the accumulation of nitrate content; the possible reason is that the gene expression of nitrate reaction is affected by the content of calcium ion [45].

Amino acids reflect the flavor and nutritional value of vegetables and are also essential components of proteins that maintain normal metabolism and growth of life. These include a class of non-protein hydrolysable amino acids called free amino acids, which can present as acidic, sweet, bitter, etc., and they are associated with the human sense of taste [47]. In this experiment, ’QYH’ with 4 mmol/L calcium treatment and ’HN’ with 6 mmol/L calcium treatment had the highest amino acid content. The reason for this may be that different cultivars of cabbage increased the absorption of macronutrients and enhanced the synthesis of amino acids by responding differently to calcium levels and concentrations. Among the 21 amino acids, the semi-essential amino acids and essential amino acids generally increased first and then decreased (except for cysteine), and there was no significant difference between cysteine and non-essential amino acids. This is somewhat similar to the findings of Broadley et al. [48].

Phenolic acids and flavonoids belong to plant secondary metabolites [49], which have multiple neutral effects such as antioxidant and anticancer [50], and they are also used as an important basis for quality evaluation. In this experiment, 12 phenolic acids and two flavonoids were detected by ultra performance liquid chromatography (UPLC), and the overall content showed a trend of increasing and then decreasing, while the flavonoids content was significantly higher than the phenolic acid content, with rutin having the highest content. There was no significant difference in the phenolic acid content and no significant difference between the two cultivars. In contrast, the phenolic acids and flavonoids contents decreased in high and insufficient calcium supply, which may be attributed to the effect on plant root growth, or related substance metabolism and signaling.

## 5. Conclusions

In conclusion, different cultivars of mini Chinese cabbage have different response to calcium concentrations; too high and too low are not conducive to the growth of mini Chinese cabbage. The appropriate calcium concentration of ’QYH’ is 6 mmol/L, while the appropriate calcium concentration of ’HN’ is 4 mmol/L. With the increase in calcium concentration, the disease index of tip-burn decreased for the accumulation of calcium in the inner leaf, and the quality of mini Chinese cabbage was improved. Our results will provide theoretical basis and technical reference for precise calcium management in the process of high-yield and high-quality production of mini Chinese cabbage.

## Figures and Tables

**Table 1:** Compound content required for different calcium level treatment.

Calcium Levels (mmol/L)	Ammonium Nitrate (mg/L)	Amount of Calcium Chloride (mg/L)	Calcium Nitrate Tetrahydrate (mg/L)	Potassium Nitrate (mg/L)	Potassium Dihydrogen Phosphate (mg/L)	Magnesium Sulfate Heptahydrate (mg/L)
0	400.217	0	0	505.5	136.09	492.94
2	240.1302	0	472.36	505.5	136.09	492.94
4	80.0434	0	944.72	505.5	136.09	492.94
6	0	111	1180.9	505.5	136.09	492.94
8	0	333	1180.9	505.5	136.09	492.94

**Table 2:** Rating of tip-burn severity in Chinese cabbage.

The Grade Standards of Tip-Burn Symptoms in Heading Chinese Cabbage	
Rating	Description of Symptoms
0	Asymptomatic
0.5	Only small spots on the edge of true leaves
1	The edge of a leaf is chlorosis
3	The edges of two leaves are chlorosis and wrinkled
5	The edges of more than two leaves are slightly tip-burn, and the tip-burn Area accounts for less than 25% of the leaves.
7	The edges of more than two leaves are moderately tip-burn, and the tip Burn area accounts for 25–50% of the leaf area.
9	The edges of more than two leaves are severely tip-burn or the whole Plant dies, and the tip-burn area accounts for more than 50% of the leaf area
11	The edges of more than two true leaves are severely burned, and the burned area accounts for more than 50% of the leaf area. The plant is weak and short.
13	Death of whole plant caused by severe dry burning

**Table 3:** Semiessential amino acid (mg/Kg).

		Calcium Levels (mmol/L)	Arg	His	Cys	Tyr
**Cultivar**	‘QYH’	0	9224 ± 123 eB	21,953 ± 1773 cB	5.10 ± 0.69 cB	459 ± 2.33 dB
		2	14,718 ± 238 cB	35,895 ± 3071 cB	6.41 ± 0.12 bB	616 ± 9.03 cB
		4	13,099 ± 439 dB	34,102 ± 2562 cB	4.27 ± 0.25 cA	494 ± 7.28 dB
		6	24,337 ± 607 aA	119,790 ± 436 aA	8.98 ± 0.37 aB	1092 ± 12.87 aA
		8	21,514 ± 413 bB	83,377 ± 9332 bB	7.17 ± 0.39 bB	932 ± 18.01 bA
	‘HN’	0	15,989 ± 327 dA	47,137 ± 974 cdA	5.38 ± 0.73 cA	587 ± 21.89 eA
		2	14,920 ± 206 dA	37,750 ± 3935 dA	9.78 ± 1.13 abA	635 ± 2.21 dA
		4	27,091 ± 646 aA	123,370 ± 1528 aA	3.97 ± 0.80 cB	1057 ± 11.41 aA
		6	17,658 ± 291 cB	57,468 ± 5729 cB	12.87 ± 1.46 aA	819 ± 13.62 cB
		8	23,494 ± 150 bA	95,678 ± 8182 bA	8.75 ± 0.64 bA	887 ± 10.70 bB
**Statistical analysis**	*P*Cultivar		***	***	**	***
	*P*Calcium Level		***	***	**	***
	*P*Cultivar × Calcium Level		***	***	*	***

Note: Data are presented as mean ± SD (n = 3) from three independent replicates. Different lowercase letters indicate significant differences between calcium levels within the same cultivar (*p* < 0.05), and different uppercase letters indicate significant differences between cultivars at the same calcium level (*p* < 0.05). Based on Tukey’s test. Asterisks (*, **, ***) denote significant correlations at *p* < 0.05, *p* < 0.01, and *p* < 0.001 levels, respectively (two-way analysis).

**Table 4:** Essential amino acid (mg/Kg).

		Calcium Levels (mmol/L)	Val	Thr	Phe	Met	Leu	Lys	Ile	Trp
**Cultivar**	‘QYH’	0	1003 ± 6.66 eB	119 ± 2.63 dB	119 ± 1.08 dB	56.66 ± 0.62 eB	475 ± 4.22 eB	4703 ± 160 dB	905 ± 11.60 dB	140 ± 2.85 eB
		2	1270 ± 4.55 dB	181 ± 1.62 cA	185 ± 1.31 cA	83.71 ± 2.72 cB	743 ± 1.23 cB	7209 ± 263 cA	1294 ± 10.24 cB	168 ± 0.98 dA
		4	1525 ± 15.90 cB	250 ± 3.42 bB	255 ± 2.59 bB	70.62 ± 2.77 dB	593 ± 5.14 dB	5053 ± 42.94 dB	1274 ± 11.64 cB	199 ± 1.24 cB
		6	3195 ± 13.72 aA	511 ± 3.45 aA	525 ± 5.03 aB	149 ± 2.85 aA	1447 ± 43.76 aA	14,530 ± 426 aA	3056 ± 8.20 aA	427 ± 2.21 aA
		8	2688 ± 41.09 bA	528 ± 13.14 aA	537 ± 14.63 aA	105 ± 2.07 bA	1165 ± 41.13 bA	10,352 ± 389 bA	2472 ± 42.03 bB	390 ± 11.30 bA
	‘HN’	0	1535 ± 9.37 dA	182 ± 4.94 dA	191 ± 4.99 dA	79.03 ± 0.57 dA	703 ± 22.81 eA	6830 ± 250 cA	1626 ± 12.44 dA	189 ± 5.25 dA
		2	1348 ± 4.19 eA	165 ± 2.10 dB	170 ± 0.16 eB	94.83 ± 2.19 cA	793 ± 5.72 dA	7227 ± 317 cA	1479 ± 14.49 eA	174 ± 1.81 dA
		4	2997 ± 18.66 aA	584 ± 0.87 aA	595 ± 2.70 aA	125 ± 2.64 aA	1233 ± 2.31 aA	13,093 ± 607 aA	2881 ± 7.41 aA	440 ± 2.34 aA
		6	2107 ± 18.78 cB	349 ± 7.29 cB	355 ± 7.99 cB	105 ± 3.20 bB	1016 ± 21.50 cB	8011 ± 362 cB	2236 ± 32.80 cB	321 ± 5.62 cB
		8	2733 ± 22.18 bA	440 ± 7.29 bB	449 ± 10.20 bB	92.68 ± 1.47 cB	1073 ± 12.86 bA	10,092 ± 395 bA	2687 ± 22.21 bA	368 ± 7.55 bB
**Statistical analysis**	*P*Cultivar		***	***	***	***	***	**	***	***
	*P*Calcium Level		***	***	***	***	***	***	***	***
	*P*Cultivar × Calcium Level		***	***	***	***	***	***	***	***

Note: Data are presented as mean ± SD (n = 3) from three independent replicates. Different lowercase letters indicate significant differences between calcium levels within the same cultivar (*p* < 0.05), and different uppercase letters indicate significant differences between cultivars at the same calcium level (*p* < 0.05). Based on Tukey’s test. Asterisks (**, ***) denote significant correlations at *p* < 0.01, and *p* < 0.001 levels, respectively (two-way analysis).

**Table 5:** Non-essential amino acid (mg/Kg).

		Calcium Levels (mmol/L)	Asn	Pro	Ala	Gly	Ser	Glu	Asp	Cyst	Gln
**Cultivar**	‘QYH	0	785 ± 13.20 dB	734 ± 2.75 eA	5341 ± 8.03 eB	284 ± 11.87 eB	879 ± 29.65 dB	1481 ± 22.00 eB	11,205 ± 171 eB	23.97 ± 1.36 eB	622.05 ± 23.28 dB
		2	1052 ± 8.79 cB	837 ± 4.69 cA	7788 ± 43.96 dA	431 ± 17.36 dA	1403 ± 1.33 cA	2399 ± 9.81 dB	15,681 ± 251 dB	44.13 ± 2.33 dA	1074.99 ± 21.47 cA
		4	1109 ± 22.40 cB	780 ± 1.74 dB	9743 ± 58.65 cB	526 ± 14.56 cB	1453 ± 6.86 cB	3174 ± 58.19 c	21,538 ± 144 cB	61.45 ± 2.06 cB	745.35 ± 19.95 dB
		6	2722 ± 13.18 aA	1454 ± 7.46 aA	22,500 ± 89.47 aA	1105 ± 21.07 aA	2786 ± 6.53 aA	7286 ± 54.47 aA	43,249 ± 33.97 aA	76.51 ± 3.65 bA	1959.34 ± 70.13 aA
		8	2127 ± 86.79 bB	1254 ± 16.36 bA	18,138 ± 259 bB	912 ± 43.59 bB	2381 ± 49.45 bB	5653 ± 160 bB	35,704 ± 951 bB	103 ± 2.83 aA	1503.13 ± 49.37 bA
	‘HN’	0	1488 ± 72.49 cA	730 ± 9.45 dA	10,319 ± 200 cA	487 ± 24.72 cA	1542 ± 27.8 7cA	3107 ± 116 dA	18,639 ± 622 cA	56.39 ± 2.91 bA	867.93 ± 8.21 dA
		2	1277 ± 8.89 dA	802 ± 4.34 cB	7764 ± 62.57 dA	391 ± 16.27 dB	1274 ± 12.15 dB	2741 ± 30.16 eA	16,554 ± 212 dB	42.72 ± 2.65 cB	960.27 ± 39.12 dB
		4	2427 ± 34.88 aA	1073 ± 9.50 aA	17,677 ± 50.41 bA	1038 ± 18.21 aA	2676 ± 5.86 aA	5867 ± 13.93 cB	43,203 ± 365 aA	87.45 ± 3.73 aA	1832.22 ± 4.63 aA
		6	1967 ± 54.69 bB	913 ± 8.32 bB	17,358 ± 102 bB	879 ± 17.90 bB	2264 ± 57.91 bB	5416 ± 170b	33,325 ± 1048 bB	50.11 ± 1.54 bcB	1124.67 ± 47.38 cB
		8	2398 ± 31.59 aA	1044 ± 12.59 aB	18,918 ± 149 aA	1065 ± 37.10 aA	2609 ± 17.82 aA	6413 ± 75.64 aA	43,493 ± 626 aA	41.71 ± 3.11 cB	1354.37 ± 35.28 bB
**Statistical analysis**	*P*Cultivar		***	***	***	***	**	***	***	**	ns
	*P*Calcium Level		***	***	***	***	***	***	***	***	***
	*P*Cultivar×Calcium Level		***	***	***	***	***	***	***	***	***

Note: Data are presented as mean ± SD (n = 3) from three independent replicates. Different lowercase letters indicate significant differences between calcium levels within the same cultivar (*p* < 0.05), and different uppercase letters indicate significant differences between cultivars at the same calcium level (*p* < 0.05). Based on Tukey’s test. Asterisks (**, ***) denote significant correlations at the *p* < 0.01, and *p* < 0.001 levels, respectively (two-way analysis) ns indicates no significant difference.

## Data Availability

The original contributions presented in this study are included in the article. Further inquiries can be directed to the corresponding author.

## Abbreviations

Zn-Zinc; K-Potassium; Mg-Magnesium; Fe-Iron; Cu-Copper; Mn-Manganese; P-Phosphorus; Ca-Calcium; Val -Valine; Thr-Threonine; Phe-Phenylalanine; Met-Methionine; Leu-Leucine; Lys-Lysine; Ile-Isoleucine; Trp-Tryptophan; Asn-Asparagine; Pro-Proline; Ala-Alanine; Gly-Glycine; Ser-Serine; Glu-Glutamic acid;Asp-Aspartic acid; Cyst-Cysteine; Gln-Glutamine; Arg-Arginine; His-Histidine; Cys-Cysteine; Tyr-Tyrosine.
